# Early development of turn-taking in vocal interaction between mothers and infants

**DOI:** 10.3389/fpsyg.2015.01167

**Published:** 2015-09-04

**Authors:** Maya Gratier, Emmanuel Devouche, Bahia Guellai, Rubia Infanti, Ebru Yilmaz, Erika Parlato-Oliveira

**Affiliations:** ^1^Université Paris Ouest Nanterre La DéfenseParis, France; ^2^Université Paris DescartesParis, France; ^3^Universidade Federal de Minas GeraisBelo Horizonte, Brazil

**Keywords:** early mother–infant communication, turn-taking, protoconversation, vocal development

## Abstract

Infants are known to engage in conversation-like exchanges from the end of the second month after birth. These ‘protoconversations’ involve both turn-taking and overlapping vocalization. Previous research has shown that the temporal organization of adult–infant turn-taking sequences is similar to that of adult verbal conversation. It has also been shown that young infants adjust the quality of their vocalization in response to the quality and timing of adult vocalization. We present new evidence of turn-taking interaction in infants aged between 8 and 21 weeks based on the analysis of 176 samples of naturalistic face-to-face interactions from 51 dyads. We found high levels of latched turns as well as frequent initiation of turn-taking by infants at these ages. Our data do not support the hypothesis that turn-taking ability increases with age between 2 and 5 months but do suggest that infants are active participants in turn-taking from the earliest age and that mothers adjust turn-taking formats to infants.

## Introduction

Conversation is a complex communicative process in which visual and auditory signals are combined in a flow of turn taking. An important aspect of the temporal organization of conversation is the fluency between turns. The end of a speaker’s turn needs to be anticipated by the next speaker for the conversation to be fluent (Sacks et al., 1974). In everyday conversation, people are remarkably rapid and accurate in switching between listener and speaker roles (Magyari and De Ruiter, 2012). The gap between turns in conversation between native speakers of the same language is generally around 250 ms (De Ruiter et al., 2006; Stivers et al., 2009). Speakers predict not only when a turn will end but also the content of the turn, enabling them to respond in a well-timed and semantically appropriate manner. Computational cognitive models fail to account for the rapidity and efficiency of transitions between speakers because they have ignored the role of embodiment in predicting speaker turns (Goodwin, 1986). Experimental studies suggest that speakers rely on both lexico-syntactic information and pragmatic signals to project the end of a turn but that accurate predictions are not possible without lexical content (De Ruiter et al., 2006).

Yet a number of studies in psychology describe turn-taking interaction occurring between infants and adults as early as in the second month of life, long before any access to lexical information. A recent study conducted by Caskey et al. (2011) on premature infants showed that as early as 32–36 weeks gestational age the frequency of infant vocalization increases in the presence of a parent. Preterm infants were also found to produce reciprocal vocalizations, supporting the hypothesis that turn-taking is a precocious human ability (Caskey et al., 2011). Stivers et al. (2009) have shown the powerful cross-cultural stability of two basic rules of the turn-taking system, avoiding overlap and minimizing silence between turns. Do young infants already know these rules or do they learn them in the course of interacting with partners? Few studies offer a developmental account of turn-taking interaction in young infants, prior to language ability.

In the first weeks after birth, infants begin to produce vocalizations that are described in the literature as coos and murmurs (Oller, 2000) and that elicit emotional and motivated responses from social partners. These vocalizations are not readily associated with either positive or negative emotion, in the way that laughter or crying are (Oller et al., 2013). They are produced when infants are alert and relaxed or playful. They are frequently associated with an intent knit-brow gaze directed at the social partner and with mouth movements resembling those of speech that have been called pre-speech movements (Trevarthen, 1993). Both caregivers and naive observers interpret these vocalizations as being produced intentionally, wilfully and with effort (Bloom and Lo, 1990; Beaumont and Bloom, 1993).

The appearance of these kinds of vocalizations gives rise to vocal exchanges with adult partners that resemble conversations because they are characterized by alternating vocalizations separated by audible pauses (Bateson, 1975; Stern et al., 1975; Trevarthen, 1979; Bloom, 1988). Adults respond to the coos and murmurs of young infants with specific prosody and timing. They have been shown to closely match the acoustic qualities of infant vocalizations, producing short repetitive bouts of infant-directed speech aimed at eliciting further vocalization on the part of the infant (Papoušek and Papoušek, 1989; Gratier and Devouche, 2011). Mothers also modulate their pitch according to the perceived emotional expressions of infants (Smith and Trainor, 2008). It has also been demonstrated that infants in turn adjust the quality of their vocalization in response to adults. The kinds of vocalizations involved in vocal turn-taking with an adult have been described as having a ‘speech-like’ quality compared with vocalizations produced alone or outside of a turn-taking format (Bloom et al., 1987). Vocal interaction in the first months of life thus appears to be bidirectional and mutually regulated (Lavelli and Fogel, 2013).

Various approaches have been used to demonstrate the active role of both mothers and infants in the first half year of life in face-to-face interaction. A durational approach reveals stable timing structures in maternal behavior across vocal and kinesic modalities (Stern et al., 1977; Cossette et al., 1986). Mothers’ speech to young infants is timed in such a way as to leave room for infant response. Maternal utterances are brief (between 0.5 and 1.5 s) and followed by pauses of around 1 s, usually followed by another utterance. Pauses between utterances that are connected through repetition of form, content, or topic rarely exceed 3 s and pauses longer than 3 s generally demarcate episodes of mutual engagement (Stern et al., 1977; Stern and Gibbon, 1979). With infants aged between 2 and 4 months, the majority of maternal responses to infants occur with a 1 s latency after the signal (Keller et al., 1999) and vocalization is responded to more frequently than gaze and smiling (Van Egeren et al., 2001). Pauses between alternating vocal turns of young infants and mothers have been found to range from 500 ms to 1 s (Jaffe et al., 2001).

A related approach to studying protoconversational organization focuses on the match or cross-correlation between temporal patterns of maternal and infant vocalization in spontaneous face-to-face interaction. The fact that mothers and infants engaged in social interaction match each other for vocalization and pause duration suggests a mutual regulation of the turn-taking exchange (Beebe et al., 1985; Jaffe et al., 2001). In her pioneering study of the protoconversations of a single infant aged between 6 and 13 weeks, Bateson (1975) highlighted the bidirectionality of infant and mother vocalization by showing that the mean duration separating successive utterances is longer when the previous utterance is by self that when it is by other. Both infant and mother respond to each other faster than they repeat an utterance of their own. Despite evidence that infants are capable of selective vocalization from the age of 2 months (Delack and Fowlow, 1978; Bloom, 1990; D’Odorico and Franco, 1991), there remains some controversy over the extent to which young infants actively contribute to turn-taking exchanges and the extent to which adults construct conversational frameworks for infant vocalization.

If infants partake in a truly co-regulated turn-taking, they must have the ability to perceive the contingent relations between their own behavior and that of their partner. These contingent relations hinge on the perception of timing in social interaction. Research has shown that infants’ sensitivity to contingency changes right around the time when protoconversations appear, around the age of 2 months (Striano et al., 2005). The still-face paradigm has demonstrated that by 2 months infants react to the sudden interruption of social interaction by the mother with reduced smiling and gazing and increased fussiness and self-comforting behavior (Tronick et al., 1978). Murray and Trevarthen’s (1985, 1986) closed-circuit double-TV paradigm shows that infants as young as 6 weeks are sensitive to social contingencies. When infants and mothers are made to interact via a live closed-circuit television set up, they are able to establish mutual gaze and partake in relaxed protoconversational interaction involving imitation across multiple modalities. However, when a sequence from the previous recording is replayed to either mother or infant with a few minutes delay, each partner becomes perturbed, expressing anxiety and aloofness. A 3 s window appears to reflect contingency for a wide range of behaviors involved in spontaneous social interaction (Van Egeren et al., 2001). The timing of an infant’s response to the partner has often been taken as a demonstration of the kind of active interpersonal coordination that underlies communication between adults.

Starting around the age of 2 months, the cooing stage (Oller, 1980; Stark, 1980) is associated with a marked rise in face-to-face interaction described as primary intersubjectivity (Trevarthen, 1977; Lavelli and Fogel, 2013). By the age of 4 months, however, infants’ interest shifts from an intense involvement with other people to involvement with objects (Trevarthen and Hubley, 1978; Cohn and Tronick, 1987). In interactive object play, adults and infants become engaged in doing things together, such as exploring objects or using them in novel ways. It is not clear from the existing literature whether the shift from primary intersubjectivity to object play involves a change in the turn-taking organization of social interaction. Indeed, very few studies have focused on turn-taking in social interactions with infants involving object play or joint attention.

One longitudinal study has reported a quantitative increase in vocal turn-taking with less overlapping vocalization between 12 and 18 weeks of age (Ginsburg and Kilbourne, 1988) suggesting infants’ turn-taking competence increases around 4 months of age. Another study supporting the hypothesis that infants’ turn-taking competence increases with age was conducted by Rutter and Durkin (1987). Using both a transversal and a longitudinal design, this study focused on vocal and gaze coordination from the end of the first year to the end of the third year of life. According to these researchers, infants younger than 24 months more frequently interrupt their mothers, whereas after this age they begin to truly coordinate their vocalizations with those of the mother. They also found that infants use gaze to actively signal the end of a turn by the age of 18 months. At this age, their gaze patterns begin to resemble those used in adult conversation in that they indicate giving the floor to their interlocutor and confirm when the floor is about to be offered to them by looking up at the interlocutor at the end of a turn.

The most detailed longitudinal study of the timing of turn-taking in infancy was conducted recently by Hilbrink et al. (submitted) on 12 infants aged between 3 and 18 months. These researchers report on the prevalence of turn-taking exchanges throughout the period they studied. They also find that turn-taking organization varies little between 3, 4, and 5 months of age but that turns slow down markedly around the age of 9 months. At this age infants respond to mothers’ utterances with longer gaps whereas maternal turn-timing remains stable over time. Furthermore, this study shows that the amount of overlap in turn-taking remains constant between 3 and 5 months of age but decreases thereafter. The slowing down of turn-taking can be associated with important qualitative changes in social interaction and communicative skills such as joint attention.

The present paper assesses developmental change in turn-taking organization between the 3rd and the 5th months of life. We first aimed to explore the hypothesis that turn-taking is, from the first weeks of life, a mutually coordinated effort and then to assess developmental change and stability in the amount of overlap, duration of switching pauses (gap) between turns and length of TTSs. We thus compared spontaneous vocal interactions between mothers and infants ages between 2 and 3 months with those of mothers with 4-to-5-months-old infants.

## Materials and Methods

### Participants

Fifty one MI dyads participated in this study. Infants were aged between 8 and 21 weeks [28 boys and 23 girls aged respectively 12.8 weeks ± 3.77 (range: 8–19) and 13.2 weeks ± 3.73 (range: 8–21)], were born full-term and in good health. Out of the 51 mothers, three mothers were bilingual speakers but spoke to their infants in French. The sample was divided into two age groups: a group of ‘younger’ infants (35 8–13 weeks, mean age 10.8 weeks ± 1.54, 15 girls) and a group of ‘older’ infants (15 17–21 weeks, mean age 18.3 ± 1.24, 8 girls). The present research was approved by the university ethics committee (CCP n°1450089).

### Apparatus and Procedure

All dyads were recorded in naturalistic contexts, in their home, when infants were in a quiet alert state. Before each observation, consent forms were signed by the parents. Mothers and infants were placed in comfortable positions, facing each other. Mothers were asked to talk to their infants in their usual manner for approximately 10 min and to avoid using any toys. Video and audio recordings were made using two camcorders (Sony Handycam HDR-CX190) and a digital audio recorder (Korg Sound on Sound Unlimited Track Recorder) placed near the dyad. Only the audio recordings were used in this study.

### Acoustic Analysis

#### Selection of the Audio Samples

A total of 176 audio samples were selected [3.5 audio samples on average per infant ± 2.2 (range: 1–9). Audio sample length was on average 106.7 s ± 57.9 (range: 18–252)]. In all 90 min and 41 s of interaction were analyzed. Samples lasted on average 31 s. The samples were taken from a large corpus of audio recordings, they were the first to meet following four selection criteria:

(1) Each sample included at least one vocal contribution by the infant and no negative infant vocalizations (fuss, cry).
(2) In each sample, mothers addressed their infant directly. Sequences including song to infants were discarded.
(3) Samples were segmented based on pause duration: pauses between vocalizations that exceeded 3 s marked the end of the selected audio sample.
(4) Recording quality was optimal for all samples.

#### Software

We used Sound Analysis Pro (Tchernichovski et al., 2000) to segment the sequences and to obtain acoustic measures of individual vocalizations. Sound Analysis Pro is used primarily in studies of birdsong but has recently been used successfully to study infant vocalization (Lipkind et al., 2013). Data were exported and manipulated in Excel.

#### Coding of Vocalizations and Pauses

Based on visualization of spectrograms and audio guidance, each sequence was manually segmented into 4 types of events: maternal vocalization, infant vocalization, overlapping vocalization and pause, according to the following criteria:

(1) A vocalization (either by mother or infant) was defined as the production of vocal sound by one partner that was either continuous or included unvoiced segments of less than 300 ms. If the silent pause following an audible vocal sound was greater than 300 ms, two successive vocalizations were coded.
(2) Overlapping vocalization was coded when either mother or infant vocalized over the vocalization of the partner. The entire vocalization was coded as overlapping even if it was only partially masked by the partner’s vocalization.
(3) Pauses occurred either between two vocalizations by the same partner (within- speaker pause) or between alternating vocalizations (switching pause). Within-speaker pauses had a duration that was necessarily greater than 300 ms and lower than 3000 ms. Switching pauses could range from a few milliseconds to 3000 ms. Two alternating vocalizations were qualified as latched when the switching pause had a duration of less than 50 ms.
(4) Vegetative sounds produced by infants such as burps, growls or hiccups, noise from the environment and vegetative sounds produced by mothers, such as coughs, were not coded.

#### Coding of Turn-Taking Sequences

A TTS was defined as sequence of vocalizations involving at least one alternation between speakers. Such a sequence could involve alternation between a vocalization of mother and of infant or between a vocalization of mother or infant and an overlapping vocalization. A TTS ended when the same speaker produced at least two vocalizations in succession or the pause following a vocalization was greater than 3000 ms.

#### Acoustic Measures

Sound Analysis Pro (Tchernichovski, 2012) provides automated analysis of various acoustic features of the segmented sounds. These include duration, amplitude, pitch, frequency modulation, and entropy. For this study only durations and frequencies of vocalizations and pauses were used.

#### Inter-Coder Reliability

Twenty percent of the data set were double-coded. Inter-coder reliability (Pearson product-moment correlations) was 0.87, 0.98, 0.78, and 0.95 respectively for number of infants’ vocalizations, mothers’ vocalizations, vocalizations involving overlap and pauses. Onset positions were considered identical if they occurred within 50 ms, thus measures of vocalization duration had an error tolerance of up to 100 ms. Both coders correctly identified 80.6% of all onset positions within the subset of double-coded sequences.

### Statistics

Analysis was done with Stata for Windows (version 12). Multiple regression was used to compute partial regression coefficients and logistic regression to estimate ORs and Wald 95% CIs (Cohen et al., 2003). Age (8–13 weeks vs. older than 13 weeks) and gender were treated as binary variables and included in the model as factors, and dyads as potential confounders. A general linear model (GLM) was used to analyze number of vocalizations per minute, with infants’ gender and age as factors and including partial eta square as index of effect size. A chi square test was applied on the contingency tables, including Cramer’s V as an index of effect size. An alpha level of 0.05 was used for all statistical tests.

## Results

### Vocal Production

The entire sample comprised a total of 2943 vocalizations of which 748 were produced by the infants alone, 1851 were produced by the mothers alone and 344 involved both mothers and infants vocalizing in overlap. Thus, the total sample of infant vocalizations comprised 1092 vocalizations and the total sample of maternal vocalizations comprises 2195 vocalizations. In total 2152 pauses were identified, of which 838 were switching pauses (38.9%).

#### Infant Vocalizations

Infant vocalizations occurring within less than 3 s of a maternal vocalization, i.e., involved in turn taking, represented 73.1% of all infant vocalizations (see **Table 1**). Infant vocalizations which were not involved in turn taking were produced either after an infant vocalization (12.8%) or were isolated, i.e., were neither preceded nor followed by another vocalization (14.1%). The proportion of infant vocalization occurring within TTSs was higher among younger infants than among older infants (78.7% vs. 62.2%; *OR* = 2.25, 95% CI: 1.69 2.99, *p* < 0.0001).

**Table 1:** Proportions of infant vocalizations according to position in relation to other vocalizations and pauses.

	Infant vocalizations (*N* = 1092)					
	All infants (*N* = 1092)		8–13 weeks (*N* = 719)		17–21 weeks (*N* = 373)	
	*N*	%	*N*	%	*N*	%
Vocalizations preceded by another infant vocalization	140	12.8%	66	9.2%	74	19.8%
**Vocalizations involved in turn taking**
Preceded by mother vocalization with pause	252	23.1%	195	27.1%	57	15.3%
Preceded by mother vocalization without pause	202	18.5%	145	20.2%	57	15.3%
Involving overlap	344	31.5%	226	31.4%	118	31.6%
**Isolated vocalizations**
Neither preceded nor followed by any other vocalization	154	14.1%	87	12.1%	67	18.0%

**FIGURE 1:**
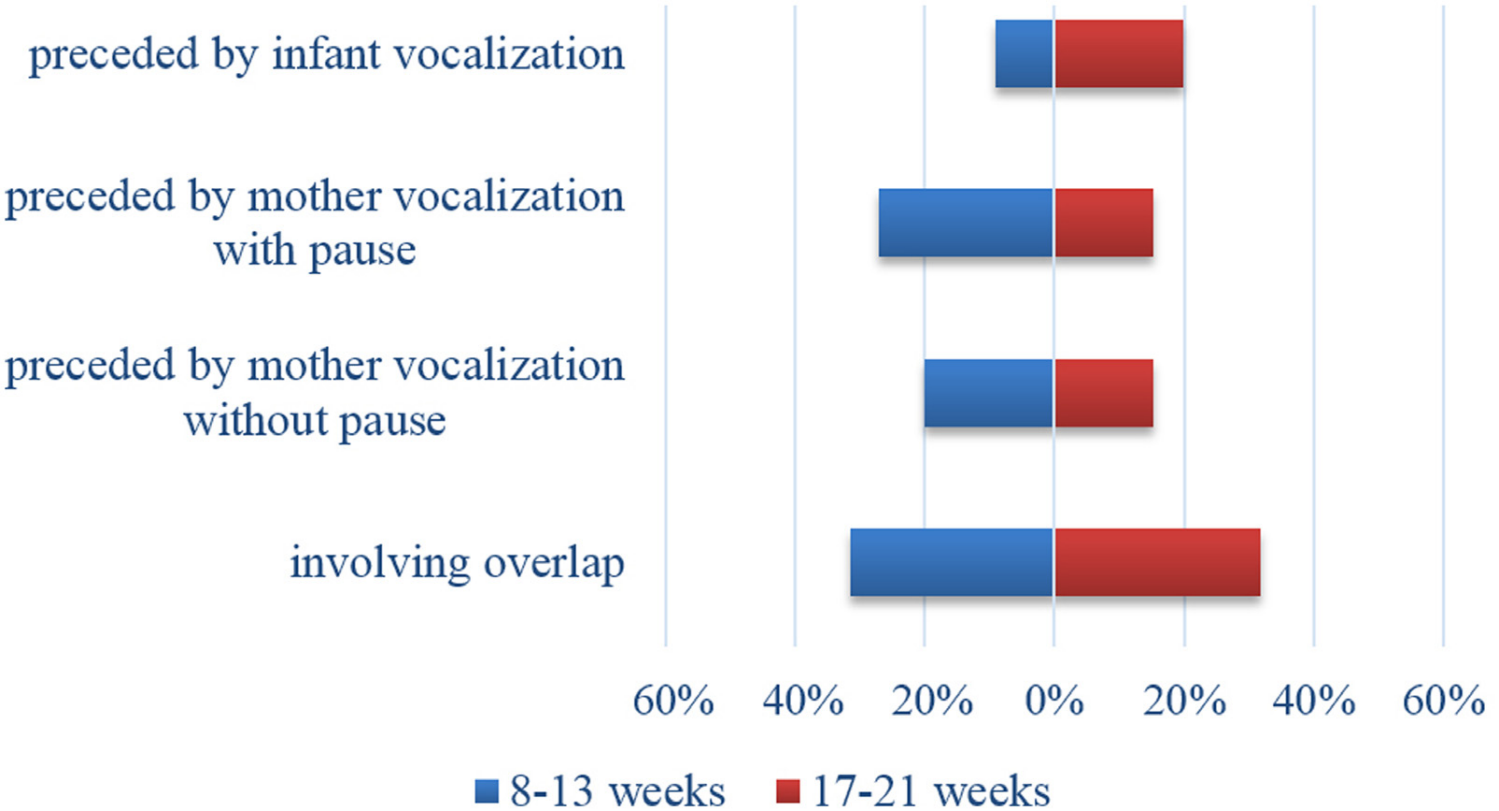
**Proportions of infant vocalization according to age**.

Among the vocalizations occurring within TTSs, some were overlapping vocalizations (*n* = 344; 31.5%), and the remaining were vocalizations that followed a maternal vocalization (*n* = 454; 41.6%). These were either latched vocalizations, that is vocalizations occurring without an intervening pause (*n* = 202; 18.5%) or vocalizations involving a switching pause (*n* = 252; 23.1%; see **Table 1**). Contingency analysis conducted on type of vocalization revealed a significant age effect (Chi square = 39.8; *p* < 0.0001; Cramer’s *V* = 0.21): older infants vocalized twice in succession more frequently than younger infants whereas vocalizations occurring after a switching pause were more frequent among the younger infants. No difference was observed between the two groups for frequency of overlapping vocalizations (see **Figure 1**).

Infant vocalizations lasted on average 869.7 ms ± 662.3 (range: 50.2–3640.0). Multiple regression conducted on the durations of vocalizations revealed no effect of age (*p* = 0.49), but a significant gender effect: girls’ vocalizations were on average 108 ms longer than boys’ vocalizations (*p* = 0.049).

#### Maternal Vocalizations

Maternal vocalizations occurring within less than 3 s of an infant vocalization – i.e., involved in turn taking – represented 35.8% of all maternal vocalizations (see **Table 2**). One in five mother vocalizations was consecutive to an infant vocalization (441; 20%), either as a latched turn (*n* = 184; 8%) or involving a switching pause (*n* = 257; 12%). Only 13.5% of mother vocalizations were not embedded in a sequence of vocalizations (*n* = 297) and 51% were preceded by a maternal vocalization (*n* = 1113).

**Table 2:** Proportions of maternal vocalizations according to position in relation to other vocalizations and pauses.

	Mother vocalizations (*N* = 2195)			
	All mothers (*N* = 2195)		8–13 weeks (*N* = 1704)		17–21 weeks (*N* = 491)	
	*N*	%	*N*	%	*N*	%
Vocalizations preceded by another mother vocalization	1113	50.7%	928	54.5%	185	37.7%
**Vocalizations involved in turn taking**
Preceded by infant vocalization with pause	257	11.7%	202	11.9%	55	11.2%
Preceded by infant vocalization without pause	184	8.4%	136	8.0%	48	9.8%
Involving overlap	344	15.7%	226	13.3%	118	24.0%
**Isolated vocalizations**
Neither preceded nor followed by any other vocalization	297	13.5%	212	12.4%	85	17.3%

**FIGURE 2:**
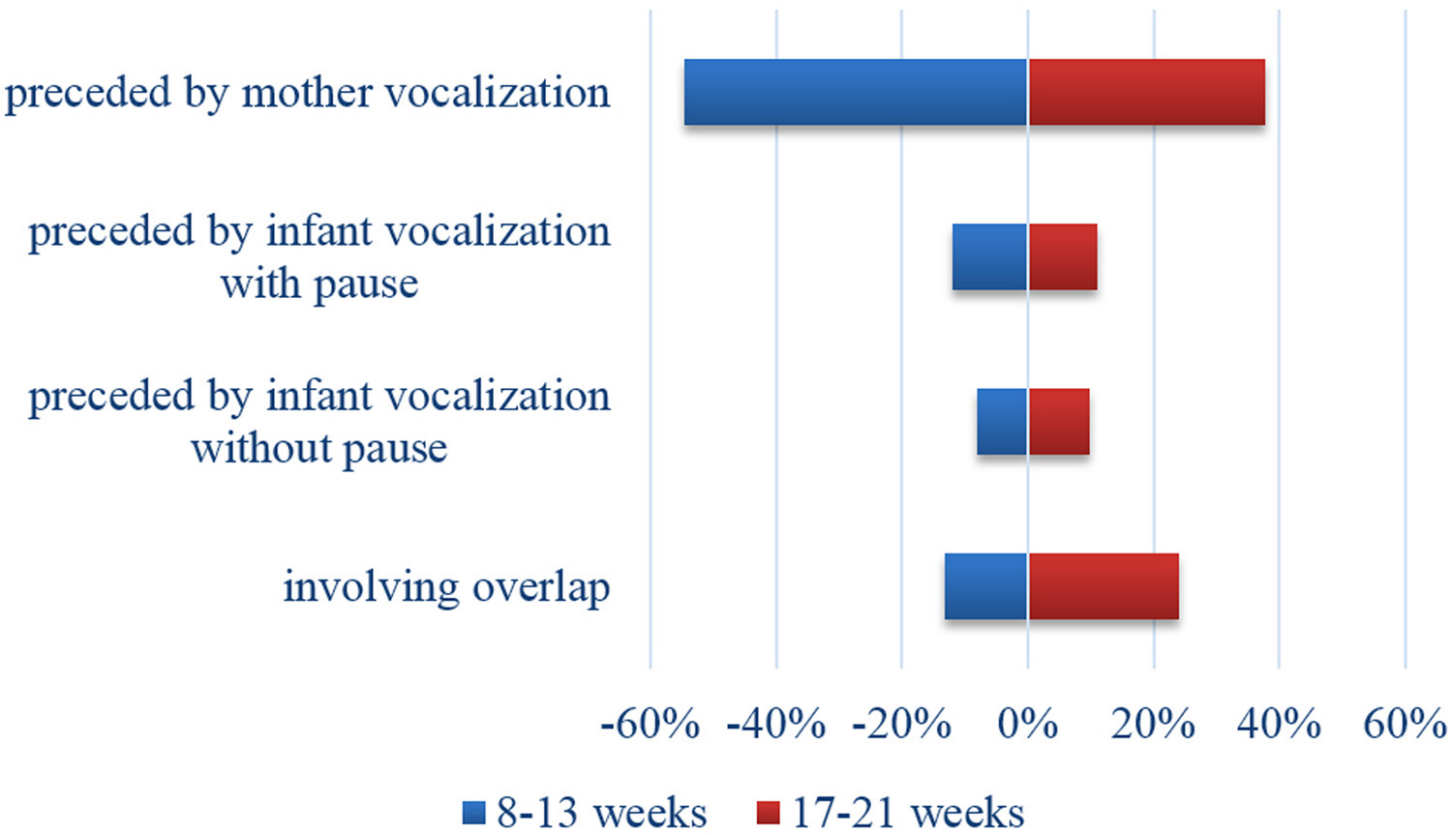
**Proportions of maternal vocalization according to infant age**.

Contingency analysis conducted on type of vocalization revealed a significant age effect (Chi square = 51.6; *p* < 0.0001; Cramer’s *V* = 0.16). Mothers vocalized twice in succession more frequently with younger infants but overlap was more frequent for mothers of older infants (see **Figure 2**).

Mothers’ vocalizations lasted on average 1144.7 ms ± 904.9 (range: 50–9803). Multiple regression conducted on the durations of the vocalizations showed a significant effect of age: the vocalizations of mothers of younger infants were on average 399 ms longer than those of mothers of older infants (*p* < 0.0001). A gender effect was also observed, mothers of boys vocalizing on average longer (*p* = 0.034).

#### Vocalizations Involving Overlap

Vocalizations involving overlap lasted on average 1550.7 ms ± 1090.1 (range: 110–4928). Multiple regression conducted on the durations of the 344 overlapping vocalizations revealed a significant effect of age: overlapping vocalizations involving younger infants were 812 ms longer than those involving older infants (*p* < 0.0001). No gender effect was found (*p* = 0.25). Analysis of the number of vocalizations per minute revealed no significant effects, neither for gender (*p* = 0.96) nor for age (*p* = 0.92).

#### Latched Turns

Among the 454 turns produced by infants 44.5% (*n* = 202) were latched, i.e., without a pause between alternating vocalizations. This proportion was quantitatively higher for older infants than for younger ones but not significantly so (50.0% vs. 42.6%; *OR* = 1.34, 95% CI: 0.86 2.10, *p* = 0.19). Maternal vocalizations preceding a latched turn by the infant were shorter, though not significantly so, than those involved in turns including a switching pause (1089 ms vs. 1152 ms; *p* = 0.35). Among the 441 turns performed by mothers, 41.7% were latched. This proportion was quantitatively higher when mothers responded to older infants than when they responded to younger infants, but again not significantly so (46.6% vs. 40.2%; *OR* = 1.29, 95% CI: 0.81 2.07, *p* = 0.26). Infant vocalizations preceding a latched turn by the mother had the same duration as those involved in turns including a switching pause (respectively 869 and 870 ms).

### Pauses

#### Within-Speaker Pauses

Pauses between vocalizations by the same speaker, which were by definition restricted to the range of 300 to 3000 ms, lasted on average 745.5 ms ± 557.0 (range: 310–2643.6) for infants and 967.3 ms ± 606.4 (range: 300–2994.8) for mothers. Multiple regression analysis revealed that within speaker pauses were not significantly different according to age, neither for infant (*p* = 0.82) or for mothers (*p* = 0.60). No gender effect was found.

#### Switching Pauses

The samples comprised 838 switching pauses lasting on average 730 ms ± 543.6 (range: 50–2974). Multiple regression analysis showed that switching pauses were on average 174.0 ms longer in samples involving older infants (*p* = 0.007). No significant gender effect was found (*p* = 0.16).

Among the switching pauses, 60.5% concerned pairs of vocalizations that were either between an IM pair of vocalizations (30.1%) or between a MI pair of vocalization (30.4%), i.e., switching pauses which did not involve an overlapping vocalization. Both types of switching pause were analyzed separately. Multiple regression analysis confirmed the overall age effect for all switching pauses (*p* = 0.013) and showed that switching pauses inside an IM pair of vocalizations were on average 135.4 ms shorter than those inside a MI pair of vocalizations (*p* = 0.014). This difference was not impacted by infant age (*p* = 0.68).

### Turn-Taking Sequences

In total, 489 TTSs were identified, lasting on average 5.68 s ± 4.39 (range: 0.3–28.2) and ranging from 2 to 18 turns (mode of three turns, i.e., three alternating vocalizations). Because duration and number of turns were highly correlated [*r* = 0.81; *t*(487) = 30.6, *p* < 0.0001], both measures were analyzed separately in order to appreciate whether they were likely to be explained by age.

**FIGURE 3:**
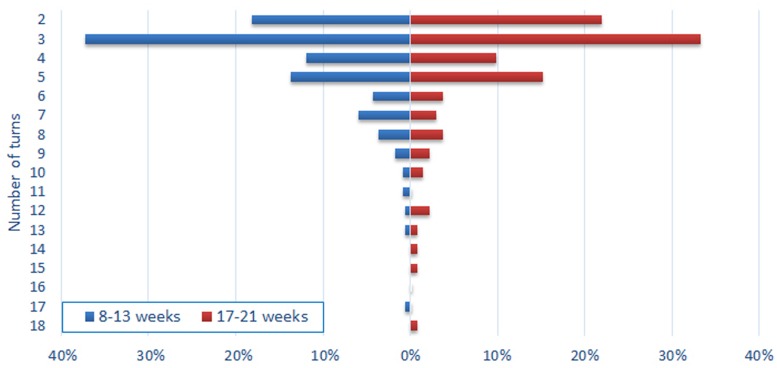
**Distribution of turn-taking sequences according to age and number of turns**.

Multiple regression conducted on the number of turns per TTS did not reveal any significant effect, neither of age (*p* = 0.57) nor of gender (*p* = 0.56). However, we did observe a significant difference between both age groups regarding the duration of TTSs: TTSs of older infants lasted 1.22 s longer (*p* = 0.005; see **Figure 3**). No significant gender effect was found (*p* = 0.53).

TTSs were divided into two subgroups depending on which partner initiated the sequence. Sequence initiation was coded when a vocalization occurring after a pause lasting at least 3 s was followed by at least one vocalization by the partner within less than 3 s. Among the 489 TTSs collected, 44.8% were initiated by infants. Logistic regression showed that TTS initiation by infant was most likely to occur in older infants (*p* = 0.003; *OR* = 1.8, 95% CI: 1.22 2.77). No significant gender effect was found (*p* = 0.30).

Turn-taking sequences were also divided in two subgroups according to which partner terminated the sequence. A sequence termination was coded when a vocalization following at least one vocalization by the partner within less than 3 s was followed by a pause of at least 3 s. Among the 489 TTSs observed, 37 (7.6%) ended with a co-vocalization and were then excluded from analysis. Twenty four percent of the remaining 447 TTSs were ended by infants. Logistic regression showed that TTSs were more likely to be ended by infants in older infants (*p* = 0.004; *OR* = 2.0, 95% CI: 1.26 3.23). We also observed a significant gender effect: boys were less likely to end a TTS than girls (*p* = 0.009; *OR* = 0.55, 95% CI: 0.35 0.86).

## Discussion

In the present study, durational features of vocal interactions between mothers and infants from two age groups (2–3 and 4–5 months) were collected using acoustic analysis software. The aim of the study was, first, to ascertain whether vocal exchanges involving young infants has a turn-taking format and, second, to investigate developmental change in vocal turn-taking.

The first finding was that three out of four infant vocalizations are either followed or preceded by a maternal vocalization. Infants in both age groups vocalize in turn as frequently, and the most common turn-taking format at both ages is one involving three turns (two alternations between mother and infant). Indeed, infants respond to mothers’ vocalizations as often as mothers respond to infants’ vocalizations at both ages. Although the turn-taking format is observed in vocal interactions of infants aged between 2 and 5 months, the question that needs addressing is whether mothers are responsible for creating these formats or whether infants actively partake in shaping TTSs.

One way to investigate infants’ active role in turn-taking is to ask whether they demonstrate anticipation of the mothers’ turns and whether they show initiative in generating a vocal response from the mother. The present study provides partial answers to these questions because it is based on durational measures and does not include other acoustic features such as intonation contour, pitch matching or amplitude modulation. Some of our findings support the hypothesis that, already from the 2nd month of life, infants anticipate turns and initiate TTSs.

Infants at both ages used a high proportion of latched turns, that is they began to vocalize within a few milliseconds (less than 50 ms) of the end of the mother’s vocalization, without any overlap. To our knowledge, no study of early MI vocal interaction has reported on the frequency or significance of latched turns. This finding is significant in teasing apart the roles of adults and infants in turn-taking exchanges. Indeed, although latched turns are less frequent than overlapping vocalization, it seems implausible that they would occur randomly at the frequencies we report. The temporal window for initiating vocalization is markedly smaller for latched turns than for turns involving a pause and for overlapping vocalizations. Indeed, the average time frame within which an infant vocalization is described as an ‘overlapping vocalization’ in our study is 1145 ms (the average duration of a maternal vocalization) and the average time frame within which an infant vocalization is involved in a turn (excluding latched turns) is 730 ms (the average duration of a switching pause). The high frequency of latched infant vocalizations suggests that infants actively shape the turn-taking organization of an exchange and anticipate events within it. Based on a comparison of the probabilities of infants producing different prosodic contours, a previous study showed that 3-months-old selectively match their vocalizations to preceding maternal vocalization (Gratier and Devouche, 2011). Thus the low probability that an infant should vocalize at a particular moment in time or in a particular way can be interpreted as a high probability that the infant vocalizes with purpose.

The finding that infants in the 3rd months already frequently perform latched turns poses a major challenge. How do young infants project the end of a turn as precisely as adults do in verbal conversation, with ‘no-gap-no-overlap’ (Sacks et al., 1974), without any lexico-syntactic information? Much research has shown that the projection of turn completion relies principally on linguistic cues (De Ruiter et al., 2006). It is possible that infants rely on durational cues to project the end of the mother’s turn and that mothers too rely on such cues to project the end of the infant’s vocalization. Maternal speech to infants is known to be rhythmic, and maternal utterances are usually short and bound by pauses. In our study, as in previous studies (Jaffe et al., 2001), maternal vocalizations lasted around 1 s. We wondered whether infants might learn their mothers’ specific time-signature which would enable them to predict with high precision when most of her utterances end. However, the duration of the vocalization to which an infant vocalization is latched does not appear to sufficiently explain the occurrence of latching. Maternal vocalizations that preceded a latched turn were only marginally longer that those preceding a switching pause. Another plausible explanation for the high percentage of latched turns is that mothers interrupt their utterances as soon as they perceive the infant is about to vocalize. Mothers may use cues such as in-breath or change in muscle tone to predict the onset of an infant vocalization. However, if it were the case that mothers interrupt their utterances rather than that infants predict the end of the mother’s utterance, maternal utterances preceding a latched infant vocalization should have been found to be shorter than maternal utterances preceding a switching pause. It is worth investigating whether other acoustic features might afford turn latching, such as drop in fundamental frequency and/or intensity. A more complete analysis of acoustic features of vocalization might provide greater insight into this phenomenon.

Furthermore, it is interesting to explore the possible functions of latched turns in protoconversational exchange. The experience of a seamless transition between self-expression and other-expression may be highly relevant for a young infant, reinforcing an emerging sense of agency in the 1st months of life. Latched turns may be conceived as joint action, where each individual’s actions are coordinated so as to achieve a joint outcome and where each individual’s action cannot be understood in isolation from the others’ (Sebanz et al., 2006).

Overall, comparison of the two age groups revealed both continuity and change in turn-taking organization. We do not find any differences in terms of mean frequency of vocalizations involving overlap between the two ages. Nor do we find that the duration of switching pauses decreases with age. On the contrary in our data they are longer among the older infants. Older infants do not perform proportionately more latched turns than younger infants. We did find that switching pauses were more frequent in younger infants’ interactions. This finding should entail that younger infants partake in a greater number of alternating vocalizations but we did not find that the number of turns within TTSs increases with age. This inconsistency may be explained by the quantification method we used for turn-taking. Overlapping vocalizations and latched vocalizations were taken into account in the quantification of number of turns whereas the frequency of switching pauses was quantified on the basis of non-overlapping vocalizations alone. It is therefore not possible to describe the contribution of each type of vocalization within the TTSs. Future studies should be aimed at more precise description of overlapping vocalization in MI interaction.

Turn-taking sequences were found to be longer for older infants despite the fact that infant vocalizations have similar durations in both age groups and that mothers’ vocalizations are shorter in interactions with older infants. Longer TTSs at 4–5 months than at 2–3 months are most likely explained by the increase in switching pause duration between the two ages. Furthermore, a greater number of infant vocalizations were either responded to or were responses to the mother among the younger infants, and older infants vocalized more often twice in succession. These two age-related changes may reflect a transition between the highly social cooing stage and the more solitary exploratory sound play that follows in vocal development (Oller, 2000). Hilbrink et al. (submitted) related their finding that turn-timing slows down around 9 months to the emergence of socio-cognitive skills that are crucial for communication and language, such as joint attention and cooperative cognition. In the present study too the younger infants responded to maternal vocalization faster than the older infants did. Similarly, this finding may be explained by a reorganization of social attention that is known to occur around 4 months, when infants become increasingly interested in object play and begin to show shared attention.

Our findings confirm the active role of mothers in adjusting their vocal behavior to infants. Mothers’ vocalizations were found to be longer with younger infants, and they produce more successive vocal utterances when interacting with younger infants. Mothers perform more latched turns with older infants and switching pauses between IM pairs of vocalizations are shorter on average than between MI pairs, suggesting that mothers respond to infants faster than infants respond to mothers. In a previous study, the duration of switching pauses in vocal turn-taking between 2 and 3 months-old infants and mothers was found to vary cross-culturally in relation with parenting styles and cultural representations (Gratier, 2003). Switching pause duration may thus reflect mothers’ representation of the infant’s communicative ability more than the infants’ turn-taking competence. We also found that mothers’ vocalizations are longer with boys than with girls confirming that gender-based representations impact infant-directed speech (Kitamura and Burnham, 2003).

The significance of our findings on initiation and termination of TTSs is ambiguous. We found that older infants were more likely to initiate a TTS. However, initiating a sequence does not imply that older infants show greater initiative in turn-taking, as this finding could be explained by mothers responding more often to the vocalizations of older than of younger infants. In fact, a recent study has shown that, without knowledge of infant age, naïve listeners implicitly attribute greater communicative intentionality to vocalizations of 5 months-old infants than to those of 1 month-old (Gratier et al., submitted). Our data also show that older infants more frequently terminate TTSs than younger infants. This may also be seen to reflect the mother’s stance toward the infant rather than a change in the way infants organize turn-taking because mothers may not respond as often to terminal vocalizations with older infants. It would be interesting to take into account the acoustic characteristics of these initial and terminal infant vocalizations in order to gain insight into the question of infant initiative. Prosodic cues such as intonation contour may signal initiative to mothers, for both initiating and terminating turn-taking. It is also possible that mothers do not respond to terminal vocalizations because the TTS has reached a durational threshold, her unresponsiveness would then serve a regulatory function. It is worth investigating the transformations of the two partners’ roles in turn-taking across ages. Indeed, mothers may progressively give the infant more and more prominence in the interaction, considering her more and more as an active partner with “something to say” while making the rules of turn-taking more salient. Infants may become less interested in a vocal turn-taking and more interested in multimodal turn-taking around object play or shared activities.

Overall our findings do not clearly support the hypothesis that infants’ become more competent at turn-taking between the 3rd and 5th months of life. Indeed, we find neither shorter switching pause durations nor less overlap between these ages. Rather, our findings suggest that turn-taking organization is sensitive to changes in infants’ social motives, reflecting a growing involvement in object play and joint activity. Although mothers are clearly highly adaptive and active in vocal exchanges with infants, we have found evidence that already very young infants play an active role in shaping the unfolding of TTSs. This exploratory study on the early development of turn-taking points to the possibility that turn-taking in preverbal interaction adapts to infants’ changing motives for communicating and learning, and paves the road to the crucial socio-cognitive skills that precede and enable language use.

## Conflict of Interest Statement

The authors declare that the research was conducted in the absence of any commercial or financial relationships that could be construed as a potential conflict of interest.

## ABBREVIATIONS

CI: confidence interval
IM: infant–mother
MI: mother–infant
OR: odds ratio
TTS: turn-taking sequence

## References

[B1] Bateson M. C. (1975). Mother-infant exchanges: the epigenesis of conversational interaction. *Ann. N. Y. Acad. Sci.* 263 101–113. 10.1111/j.1749-6632.1975.tb41575.x 1060428

[B2] Beaumont S. L. Bloom K. (1993). Adults’ attributions of intentionality to vocalizing infants. *First Lang.* 13 235–247. 10.1177/014272379301303805

[B3] Beebe B. Jaffe J. Feldstein S. Mays K. Alson D. (1985). “Interpersonal timing: the application of an adult dialogue model to mother-infant vocal and kinesic interactions,” in *Social Perception in Infants* eds Field T. Fox N. (Norwood, NJ: Ablex Publishing Corporation) 217–247.

[B4] Bloom K. (1988). Quality of adult vocalizations affects the quality of infant vocalizations. *J. Child Lang.* 15 469–480. 10.1017/S0305000900012502 3198716

[B5] Bloom K. Lo E. (1990). Adult perceptions of vocalizing infants. *Infant Behav. Dev.* 13 209–219. 10.1016/0163-6383(90)90031-3

[B6] Bloom K. Russell A. Wassenberg K. (1987). Turn taking affects the quality of infant vocalizations. *J. Child Lang.* 14 211–227. 10.1017/S0305000900012897 3611239

[B7] Bloom L. (1990). “Developments in expression: affect and speech,” in *Psychological and Biological Approaches to Emotion*, eds Stein N. L. Leventhal B. Trabasso T. (Hillsdale, NJ: Lawrence Erlbaum Associates, Inc.) 215–246.

[B8] Caskey M. Stephens B. Tucker R. Vohr B. (2011). Importance of parent talk on the development of preterm infant vocalizations. *Pediatrics* 128 910–916. 10.1542/peds.2011-0609 22007020

[B9] Cohen J. Cohen P. West S. G. Aiken L. S. (2003). *Applied Multiple Regression/Correlation Analysis for the Behavioral Sciences* 3rd Edn. Mahwah, NJ: Lawrence Erlbaum Associates, Publishers.

[B10] Cohn J. E. Tronick E. (1987). Mother-infant face-to-face interaction: the sequence of dyadic states at 3, 6, and 9 months. *Dev. Psychol.* 23 68–77. 10.1037/0012-1649.23.1.68

[B11] Cossette L. Malcuit G. Pomerleau A. Julien D. (1986). Temporal structure of maternal language directed at infants 3 months old. *Can. J. Psychol.* 40 414–422. 10.1037/h0080106 3502880

[B12] Delack L. B. Fowlow P. J. (1978). “The ontogenesis of different vocalizations: development of prosodic contrastivity during the first year of life,” in *The Development of Communication* eds Waterson N. Snow C. (London: Wiley) 93–110.

[B13] De Ruiter J. P. Mitterer H. Enfield N. J. (2006). Projecting the end of a speaker’s turn: a cognitive cornerstone of conversation. *Language* 82 515–535. 10.1353/lan.2006.0130

[B14] D’Odorico L. Franco F. (1991). Selective production of vocalizations in different communication contexts. *J. Child Lang.* 18 475–499. 10.1017/S0305000900011211 1761610

[B15] Ginsburg G. P. Kilbourne B. K. (1988). Emergence of vocal alternation in mother-infant interchanges. *J. Child Lang.* 15 221–235. 10.1017/S0305000900012344 3209641

[B16] Goodwin C. (1986). Gestures as a resource for the organization of mutual orientation. *Semiotica* 62 29–49. 10.1515/semi.1986.62.1-2.29

[B17] Gratier M. (2003). Expressive timing and interactional synchrony between mothers and infants: cultural similarities, cultural differences, and the immigration experience. *Cogn. Dev.* 18 533–554. 10.1016/j.cogdev.2003.09.009

[B18] Gratier M. Devouche E. (2011). Imitation and repetition of prosodic contour in vocal interaction at 3 months. *Dev. Psychol.* 47 67–76. 10.1037/a0020722 21244150

[B19] Jaffe J. Beebe B. Feldstein S. Crown C. L. Jasnow M. D. Rochat P. (2001). Rhythms of dialogue in infancy: coordinated timing in development. *Monogr. Soc. Res. Child Dev.* 66 1–8. 10.1111/1540-5834.00137 11428150

[B20] Keller H. Lohaus A. Völker S. Cappenberg M. Chasiotis A. (1999). Temporal contingency as an independent component of parenting behavior. *Child Dev.* 70 474–485. 10.1111/1467-8624.00034

[B21] Kitamura C. Burnham D. (2003). Pitch and communicative intent in mother’s speech: adjustments for age and sex in the first year. *Infancy* 4 85–110. 10.1207/S15327078IN0401_5

[B22] Lavelli M. Fogel A. (2013). Interdyad differences in early mother–infant face-to-face communication: real-time dynamics and developmental pathways. *Dev. Psychol.* 49 2257–2271. 10.1037/a0032268 23527490

[B23] Lipkind D. Marcus G. F. Bemis D. Sasahara K. Jacoby N. Takahashi M. (2013). Stepwise acquisition of vocal combinatorial capacity in songbirds and human infants. *Nature* 498 104–108. 10.1038/nature12173 23719373 PMC3676428

[B24] Magyari L. De Ruiter J. P. (2012). Prediction of turn-ends based on anticipation of upcoming words. *Front. Psychol.* 3:376 10.3389/fpsyg.2012.00376 PMC3483054 23112776

[B25] Murray L. Trevarthen C. (1985). “Emotional regulations of interactions between two-month-olds and their mothers,” in *Social Perception in Infants*, eds Field T. M. Fox N. A. (Norwood, NJ: Ablex Publishing Corporation) 177–197.

[B26] Murray L. Trevarthen C. (1986). The infant’s role in mother–infant communications. *J. Child Lang.* 13 15–29. 10.1017/S0305000900000271 3949895

[B27] Oller D. K. (1980). “The emergence of the sounds of speech in infancy,” in *Child Phonology* Vol 1 eds Yeni-Komshian G. Kavanagh J. Ferguson C. (New York, NY: Academic Press) 93–112.

[B28] Oller D. K. (2000). *The Emergence of the Speech Capacity.* Hove: Psychology Press.

[B29] Oller D. K. Buder E. H. Ramsdell H. L. Warlaumont A. S. Chorna L. Bakeman R. (2013). Functional flexibility of infant vocalization and the emergence of language. *Proc. Natl. Acad. Sci. U.S.A.* 110 6318–6323. 10.1073/pnas.1300337110 23550164 PMC3631625

[B30] Papoušek M. Papoušek H. (1989). Forms and functions of vocal matching in interactions between mothers and their precanonical infants. *First Lang.* 9 137–157. 10.1177/014272378900900603

[B31] Rutter D. R. Durkin K. (1987). Turn-taking in mother-infant interaction: an examination of vocalizations and gaze. *Dev. Psychol.* 23 54–61. 10.1037/0012-1649.23.1.54

[B32] Sacks H. Schegloff E. A. Jefferson G. (1974). A simplest systematics for the organization of turn-taking for conversation. *Language* 50 696–735. 10.1353/lan.1974.0010

[B33] Sebanz N. Bekkering H. Knoblich G. (2006). Joint action: bodies and minds moving together. *Trends Cogn. Sci.* 10 70–76. 10.1016/j.tics.2005.12.009 16406326

[B34] Smith N. A. Trainor L. J. (2008). Infant-directed speech is modulated by infant feedback. *Infancy* 13 410–420. 10.1080/15250000802188719

[B35] Stark R. E. (1980). “Stages of speech development in the first year of life,” in *Child phonology*, eds Yeni-Komshian G. Kavanagh J. Ferguson C. (New York, NY: Academic Press) 73–90.

[B36] Stern D. N. Beebe B. Jaffe J. Bennett S. L. (1977). “The infant’s stimulus world during social interaction: a study of caregiver behaviors with particular reference to repetition timing,” in *Studies in Mother–Infant Interaction* ed. Schaffer H. R. (New York, NY: Academic Press) 177–202.

[B37] Stern D. N. Gibbon J. (1979). “Temporal expectancies of social behaviours in mother-infant play,” in *Origins of the Infant*’*s Social Responsiveness* ed. Thoman E. B. (Hillsdale, NJ: Lawrence Erlbaum Associates) 409–429.

[B38] Stern D. N. Jaffe J. Beebe B. Bennett S. L. (1975). Vocalizing in unison and in alternation: two modes of communication within the mother-infant dyad. *Ann. N. Y. Acad. Sci.* 263 89–100. 10.1111/j.1749-6632.1975.tb41574.x 1060437

[B39] Stivers T. Enfield N. J. Brown P. Englert C. Hayashi M. Heinemann T. (2009). Universals and cultural variation in turn-taking in conversation. *Proc. Natl. Acad. Sci. U.S.A.* 106 10587–10592. 10.1073/pnas.0903616106 19553212 PMC2705608

[B40] Striano T. Henning A. Stahl D. (2005). Sensitivity to social contingencies between 1 and 3 months of age. *Dev. Sci.* 8 509–518. 10.1111/j.1467-7687.2005.00442.x 16246242

[B41] Tchernichovski O. (2012). *User Manuel.* Availble at: http://soundanalysispro.com

[B42] Tchernichovski O. Nottebohm F. Ho C. E. Bijan P. Mitra P. P. (2000). A procedure for an automated measurement of song similarity. *Anim. Behav.* 59 1167–1176. 10.1006/anbe.1999.1416 10877896

[B43] Trevarthen C. (1977). “Descriptive analyses of infant communicative behavior,” in *Studies in Mother-Infant Interaction* ed. Schaffer H. R. (London: Academic Press) 227–270.

[B44] Trevarthen C. (1979). “Communication and cooperation in early infancy: a description of primary intersubjectivity,” in *Before Speech: The Beginning of Interpersonal Communication* ed. Bullowa M. (Cambridge: Cambridge University Press) 321–347.

[B45] Trevarthen C. (1993). “The self-born in intersubjectivity: the psychology of an infant communicating,” in *The Perceived Self: Ecological and Interpersonal Sources of Self-Knowledge* ed. Neisser U. (New York, NY: Cambridge University Press) 121–173.

[B46] Trevarthen C. Hubley P. (1978). “Secondary intersubjectivity: confidence, confiding and acts of meaning in the first year,” in *Action, Gesture and Symbol: The Emergence of the Language* ed. Lock A. (London: Academic Press) 183–229.

[B47] Tronick E. Als H. Adamson L. Wise S. Brazelton T. B. (1978). The infant’s response to entrapment between contradictory messages in face-to-face interaction. *J. Am. Acad. Child Psychiatry* 17 1–13. 10.1016/S0002-7138(09)62273-1 632477

[B48] Van Egeren L. A. Barratt M. S. Roach M. A. (2001). Mother–infant responsiveness: timing, mutual regulation, and interactional context. *Dev. Psychol.* 37 684–697. 10.1037/0012-1649.37.5.684 11552763

